# A Study on the Efficacy of a Naloxone Training Program

**DOI:** 10.7759/cureus.19831

**Published:** 2021-11-23

**Authors:** Gillian A Beauchamp, Hoonani M Cuadrado, Seth Campbell, Bennie B Eliason, Chase L Jones, Aaron T Fedor, Lauren Grantz, Paige Roth, Marna Rayl Greenberg

**Affiliations:** 1 Emergency and Hospital Medicine, Lehigh Valley Health Network Campus/University of South Florida Morsani College of Medicine, Allentown, USA; 2 Street Medicine, Lehigh Valley Health Network Campus/University of South Florida Morsani College of Medicine, Allentown, USA; 3 Street Medicine, Valley Health Partners, Allentown, USA; 4 Pharmacy, Lehigh Valley Health Network Campus/University of South Florida Morsani College of Medicine, Allentown, USA

**Keywords:** opioid epidemic, community education, underserved populations, harm reduction, street medicine, naloxone

## Abstract

Introduction: The use of naloxone to reverse a potentially fatal opioid overdose is a harm reduction strategy that reduces mortality and increases the potential for referral to substance use treatment for affected individuals. In the setting of outreach performed by a street medicine team, we aimed to determine the effectiveness of an educational intervention involving distribution of naloxone accompanied by a brief instructive session about opioids, opioid overdose, and medication administration.

Methods: Our street medicine outreach team distributed 200 naloxone kits to clinicians and volunteers involved in caring for patients on ‘street rounds,’ as well as in shelters, soup kitchens, and street medicine clinic settings. Those receiving a naloxone kit engaged in a peer-reviewed presentation on how to safely use the medication to reverse a potentially fatal opioid overdose. The study team developed and administered a pre- and post-survey of 10 multiple choice questions on material covered in the educational training. The pre- and post-survey scores were compared to assess the effectiveness of implementing this training. Results were stratified by participant gender and age group.

Results: Out of the 200 participants, six were excluded from the analysis due to completely missing data from one or both surveys. The mean age of participants was 40.2±12.5 years; 120 (65.6%) were female, 62 (33.9%) were male, and 1 (0.6%) identified as nonbinary. Every survey question had an increase in correct responses from pre-survey to post-survey (identified by an increase in the percentage of correct responses). The mean survey total score increased from 5.5±1.6 to 7.5±1.3. Within the sample of 194, the mean difference in scores from pre-survey to post-survey was 2.02 points (95% CI [1.77, 2.26]), p<0.0001. Males had a mean increase in the total score from 5.6±1.8 to 7.4±1.1. Females had a mean increase in the total score from 5.5±1.5 to 7.5±1.3. The difference in total scores in males was 1.89 points (95% CI [1.42, 2.35]), p<0.0001, and in females was 2.02 points (95% CI [1.71, 2.32]), p<0.0001. Post-test scores improved in all age groups.

Conclusion: The educational training on opioids, opioid overdose, and the use of naloxone was an effective adjunct to naloxone kit distribution to volunteers and clinicians caring for people experiencing homelessness.

## Introduction

In the new millennium, there have already been nearly 500,000 deaths related to opioid overdose [1]. While multifactorial, the opioid overdose crisis likely began with the over-prescribing of opioids in the late 1990s. Subsequent studies demonstrated that most heroin users in the early 2000s initiated their opioid use trajectory with prescription opioids [2,3]. As people with opioid use conditions transitioned to street opioids, and as powerful synthetic opioids became more prolific within that street supply, the rate of fatal overdose rose from 1:100,000 in 2013 to nearly 9:100,000 in 2018, with some states reporting high-potency fentanyl detected in greater than 50% of all overdose-related deaths [1,4].

Despite being invented in 1961, naloxone was uncommonly prescribed until the 1990s, and even then, it was relegated to intravenous use until 2014 [5]. Once intramuscular and intranasal formulations became available, studies showed similar bio-availability, time to symptom reversal, and efficacy for intranasal formulations versus intravascular or intramuscular delivery [5-8]. Emergency medical services (EMS) professionals equipped with intranasal naloxone have achieved reversal in greater than 98% of cases of suspected or known opioid overdose [6,9]. Concerns have been raised about the use of naloxone in patients who have used long-acting opioids, who may be at higher risk of morbidity and mortality after receiving naloxone. However, a study of 552 patients who received naloxone in the field for suspected overdose found no reported fatalities among this group in the 48 hours after treatment [10].

Underutilization of naloxone may be in part due to stigma over its possession, lack of availability, as well as variability among state laws regarding layperson distribution [11-13]. In states in which naloxone availability is mandated at pharmacies, it has been found that locations in neighborhoods with lower socioeconomic status reported either lower quantity or no stock at all, potentially contributing to a lack of access in these environments [12]. Vulnerable populations in urban neighborhoods as well as communities with high rates of homelessness have reported fear of punitive legal action if naloxone is used and emergency services are activated [13].

Lack of education regarding the effects of naloxone in acute opioid overdose may further contribute to hesitation among bystanders to carry and use naloxone. Though there have been case descriptions of non-cardiogenic pulmonary edema and life-threatening dysrhythmias, the overall reported incidence of these events has been as low as 1%, with all occurrences being associated with intravenous administration [14,15]. The primary end point of titration for naloxone dosing is decreased respiratory depression; however, there may be a lay misconception that resolution of sedation is the true goal of therapy, resulting in the administration of unnecessary additional doses of naloxone that can lead to severe withdrawal symptoms. Given these concerns, it is likely that more widely available and standardized community education programs could lead to more appropriate naloxone use.

One study found that 5%-16% of patients presenting to an emergency department for medical care were experiencing homelessness [16]. According to the Substance Abuse and Mental Health Services Administration, the estimated rate of substance use disorder among persons experiencing homelessness is 26%-38% [17]. Due to the stigma surrounding both substance use disorder and homelessness, it is imperative to ensure the clinicians who serve stigmatized populations have access to naloxone and education on how to use this medication.

For community members with stigmatizing conditions such as lack of shelter and substance use disorder, mobile access to care including high-cost lifesaving medications, such as naloxone, may help save lives and ensure patients have access to the care they deserve. At our institution, the street medicine team practices a model that focuses on delivering care wherever persons with homelessness feel most comfortable, including providing access to naloxone during episodes of care in shelters, soup kitchens, and in encampments. In the setting of outreach performed by a street medicine team, we set out to determine the effectiveness of a brief educational intervention involving the distribution of naloxone accompanied by education on opioids, opioid overdose, and how to administer the medication.

Naloxone training programs have been shown to be effective in improving knowledge around overdose recognition and the use of naloxone [18]. Overdose education and naloxone distribution programs improve self-efficacy surrounding overdose prevention and response for up to 12 months following a brief training [19]. Training first responders and other clinicians with a brief training can improve their perceptions and comfort with overdose and naloxone use [20].

We hypothesized that providing naloxone kits and accompanying education to street medicine outreach clinicians and volunteer ‘first responders’ would increase knowledge around opioids, opioid overdose, and how naloxone is used, as measured by pre- and post-education testing.

Abstract presentations describing this initiative were presented in virtual format at the Pennsylvania American College of Emergency Physicians meeting on April 9, 2021, and at the Society for Academic Emergency Medicine meeting on May 13, 2021.

## Materials and methods

Our street medicine team distributed 200 naloxone kits accompanied by an educational training to a convenience sample of adult clinicians involved in street outreach, as well as to a convenience sample of volunteers and ‘first responders’ in the shelters, soup kitchens, and street medicine clinic settings. All participants were age ≥18 years. Those receiving naloxone kits also received an educational training on how to safely use the medication to reverse a potentially fatal opioid overdose.

Community sites where the street medicine team engaged regularly with this vulnerable population were offered the opportunity for training. All sites showed interest in the training program and the street medicine team collaborated via email with the study research coordinators to establish a date and time for the training. The individual sites notified their respective team members about the agreed upon training time and location; participation of trainees was voluntary, not mandatory. On arrival at the scheduled training time, potential participants were given a standard project information sheet and offered the opportunity to voluntarily participate in the naloxone education session and self-administer a brief pre- and post-survey. The training was provided by the two street medicine team physician assistants who led the program. Although there were occasional exceptions in which training was performed by their personal volunteer time, the preponderance of educational time spent by the street medicine physician assistants was woven into their workday, which is paid for by their hospital salaries.

The references cited in this article were utilized to guide the development of the training curriculum. The training was provided via a PowerPoint slide presentation that was peer-reviewed prior to its use by an addiction recovery specialist and a board-certified addiction medicine physician, both of whom volunteered their time, and have extensive experience in delivering community naloxone training.

We administered a pre- and post-survey of 10 multiple choice questions on material covered in the educational training. The pre- and post-survey questions were developed by the street medicine team. Before its use in the study, the survey was also peer-reviewed by a certified recovery specialist and a board-certified addiction medicine physician who volunteered their time. Survey scores (10 point maximum) were compared to assess any change in the number of correct responses before and after the brief educational intervention. The pre- and post-surveys were the same with the exception of optional demographic questions that were added to the pre-survey (Appendix). The pre-survey was given to participants before they received the educational training, and the post-survey was given after they received the training. A research assistant assigned a four-digit code to each participant to ensure pre- and post-surveys could be compared. The trained research assistant collected the paper surveys and entered the data into a password-protected electronic database accessible only to designated study members. The training program was provided using a laptop for the PowerPoint slide and was approximately 60 minutes in length.

Descriptive statistics were generated for the sample. Categorical variables were described using the frequency and percentage, and continuous variables were described using the mean, standard deviation, median, and interquartile ranges as appropriate. By author convention, the pre-and post-survey data was also stratified by gender and age group to determine any influence of these demographics on survey results. To compare pre-education test scores to post-education test scores, the number of correct questions were summed for each test by participant, and a paired t-test was used to determine if there was a statistically significant change in the number of correct answers. All analyses were two-tailed and alpha was set to significance at 0.05. SAS 9.4 (SAS Institute Inc., Cary, NC, USA) was used to conduct the analysis.

The data for this study was stored and maintained in Research Electronic Data Capture (REDCap). REDCap is a secure, web-based software platform designed to support data capture for research studies [21,22].

REDCap is installed directly on our institutional servers and is only accessible to individuals with an institutional REDCap account. While potentially sensitive information such as age, gender, and improvement in the number of correct survey questions was collected in the database, only those individuals listed as investigators for the study had access to it. Data exported from this database was placed in a password-protected spreadsheet for purposes of statistical analysis. This study was approved by the hospital network institutional review board.

This study was funded, in part, by an unrestricted community foundation research and development award for transformational excellence. The grant supported the naloxone purchase and research assistant time.

## Results

A total of 200 survey participants were included. Six participants were excluded due to missing responses to the pre-survey, post-survey or both pre- and post-survey questions. These six included two participants who ‘opted out’ of participation leaving 194 subjects included in the analysis. Demographics of the study participants and their training location can be found in Table 1.

**Table 1:** Demographics for the entire sample and stratified by gender (n=194) SD=standard deviation. Data are expressed as n (%) unless otherwise stated. Percentages might not add to 100% due to rounding. If a training location was written under ‘Other’ with a frequency of >3, this location was then created as a new training location category (e.g., School of Nursing).

	Total sample (n=194)	Male (n=62)	Female (n=120)
Training location			
Ripple Community Inc.	19 (9.8)	6 (9.7)	13 (10.8)
Safe Harbor Shelter	2 (1.0)	0	2 (1.7)
Trinity Soup Kitchen	7 (3.6)	4 (6.5)	3 (2.5)
Alliance Hall Soup Kitchen	4 (2.1)	1 (1.6)	2 (1.7)
Bethlehem Emergency Shelter	6 (3.1)	5 (8.1)	1 (0.8)
Allentown Warming Station	40 (20.6)	21 (33.9)	19 (15.8)
Bethlehem Recovery Center	17 (8.8)	4 (6.5)	13 (10.8)
Community Action Committee	25 (12.9)	5 (8.1)	15 (12.5)
Change on 3rd Street	17 (8.8)	8 (12.9)	8 (6.7)
Salvation Army	11 (5.7)	2 (3.2)	7 (5.8)
Highmark	7 (3.6)	2 (3.2)	3 (2.5)
School of Nursing	6 (3.1)	0	6 (5.0)
Family Health Center	16 (8.3)	1 (1.6)	14 (11.7)
Valley Health Partners	7 (3.6)	1 (1.6)	6 (5.0)
Other	10 (5.2)	2 (3.2)	8 (6.7)
Age, years (n=138), mean±SD	40.2±12.5	43.1±12.4	38.8±12.5
Age category (n=138)	(n=138)	(n=48)	(n=88)
20-29	29 (21.0)	7 (14.6)	21 (23.9)
30-39	46 (33.3)	14 (29.2)	31 (35.2)
40-49	27 (19.6)	7 (14.6)	20 (22.7)
50-59	23 (16.7)	16 (33.3)	7 (8.0)
60-69	11 (8.0)	4 (8.3)	7 (8.0)
70+	2 (1.5)	0	2 (2.3)

Not every participant responded to all survey questions. The number of responses for each question, for each survey, can be found in Table 2.

**Table 2:** Pre- and post-survey questions for the entire sample (n=194) SD=standard deviation. Data are expressed as n (%) unless otherwise stated. Percentages might not add to 100% due to rounding. Some participants were noted to have circled multiple answers; due to the nature of data collection and because only one answer could be selected, these participants were considered to have a missing answer and were also counted as incorrect. Correct answers and n (%) for those are bolded.

	Pre-survey	Post-survey
1. All opioids (e.g., fentanyl, heroin, prescription pain pills) have the potential to cause physical dependence.	(n=193)	(n=191)
True	191 (99.0)	190 (99.5)
False	2 (1.0)	1 (0.5)
2. What is the leading cause of injury-related death in the United States?	(n=191)	(n=191)
Alcohol overdose	15 (7.9)	3 (1.6)
Falls	7 (3.7)	1 (0.5)
Fire/burn	1 (0.5)	0
Motor vehicle crashes	41 (21.5)	2 (1.1)
Opioid overdose	127 (66.5)	185 (96.9)
3. What cause(s) a person to be at a higher risk of an opioid overdose?	(n=192)	(n=194)
Having a medical condition such as HIV, liver, or lung disease	0	1 (0.5)
Taking higher doses of an opioid	5 (2.6)	3 (1.6)
Taking opioids with other sedating substances	5 (2.6)	1 (0.5)
Taking opioids after reduced tolerance (detox or incarceration)	6 (3.1)	1 (0.5)
All of the above	176 (91.7)	188 (96.9)
4. What is a symptom(s) of an opioid high?	(n=192)	(n=194)
Blue fingernails or lips	1 (0.5)	1 (0.5)
Constricted pupils	37 (19.3)	42 (21.7)
Gurgling	1 (0.5)	0
Unresponsive	8 (4.2)	1 (0.5)
All of the above	145 (75.5)	150 (77.3)
5. What’s the first step in managing a suspected opioid overdose?	(n=191)	(n=193)
Begin rescue breathing	6 (3.1)	1 (0.5)
Call 911	110 (57.6)	181 (93.8)
Give naloxone intra-nasally	45 (23.6)	6 (3.1)
Lay person in recovery position	15 (7.9)	1 (0.5)
Lay person on back	15 (7.9)	4 (2.1)
6. Administration of naloxone should be continued every minute until patient is revived.	(n=183)	(n=191)
True	51 (27.9)	7 (3.7)
False	132 (72.1)	184 (96.3)
7. After the initial administration of naloxone, what is the next step?	(n=184)	(n=193)
Begin hands-only cardiopulmonary resuscitation (CPR)	33 (17.9)	6 (3.1)
Call 911	54 (29.4)	14 (7.3)
Do not leave person	21 (11.4)	8 (4.2)
Head tilt/jaw-thrust	12 (6.5)	12 (6.2)
Lay person in recovery position	64 (34.8)	153 (79.3)
8. Which symptom(s) is indicative of opioid withdrawal?	(n=191)	(n=193)
Body aches	20 (10.5)	40 (20.7)
Dry skin	2 (1.1)	0
Chest pain	0	2 (1.0)
Sluggishness	2 (1.1)	5 (2.6)
All of the above	167 (87.4)	146 (75.7)
9. Narcan is effective for reversing which of the following overdoses?	(n=181)	(n=187)
Benzodiazepines	28 (15.5)	9 (4.8)
Cocaine	11 (6.1)	2 (1.1)
Fentanyl	111 (61.3)	167 (89.3)
K2	2 (1.1)	2 (1.1)
Methamphetamine	29 (16.0)	7 (3.7)
10. The Good Samaritan Law only protects lay persons when responding to an opioid overdose and does not provide the person seeking medical attention after an overdose immunity from arrest.	(n=187)	(n=190)
True	95 (50.8)	69 (36.3)
False	92 (49.2)	121 (63.7)
Total score (range 0-10), mean±SD	5.5±1.6	7.5±1.3

Every survey question saw an increase in correct responses from pre-survey to post-survey (identified by an increase in the percentage of correct responses). The mean total score increased from 5.5±1.6 to 7.5±1.3. Within the sample of 194, the mean difference in scores from pre-survey to post-survey was 2.02 points (95% CI [1.77, 2.26]); this finding was statistically significant (p<0.0001).

**Figure 1:**
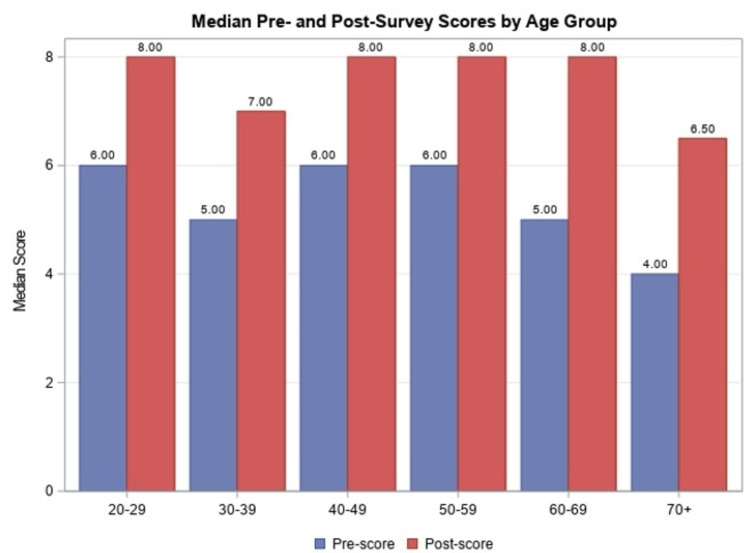
Median pre- and post-survey scores by age group

Males had a mean increase in the total score from 5.6±1.8 to 7.4±1.1. In the female sub-group, all survey questions had an increase in the percentage of participants who chose the correct answer response, with a mean increase in the total score from 5.5±1.5 to 7.5±1.3. There were no statistically significant differences found in pre-survey scores, post-survey scores, or difference in total scores between males and females. A comparison of mean pre- and post-survey scores by age group can be found in Figure 1.

## Discussion

This study demonstrated that a brief educational presentation regarding naloxone, opioids, and overdose, resulted in an increase in correct post-intervention survey responses regardless of the participant age or sex. This study contributes to the literature by highlighting the effectiveness of implementing a brief educational intervention to accompany naloxone kit distribution to a small sample of people caring for individuals experiencing homelessness.

We were able to successfully provide the educational training at over 15 different types of sites (soup kitchens, shelters, etc). The number of the participants at each site was too small to do a statistical comparison or sub-analysis for performance on the pre-/post-tests by site. This is an opportunity for future research to glean a better understanding of how site-specific differences could impact the results.

Additionally, there were variations in the frequency of correct responses for individual questions. There were some that most people got right and a few that appear to have been poorly understood before the training. For instance, while nearly everyone understood that opioids could be addictive, their lethality or how to recognize and intervene effectively for an overdose victim were not as universally comprehended. This underscores the need for public health interventions and education of our community in this regard.

Some studies evaluating naloxone education focus on participants with a history of opioid use. Barocas et al. found that of 543 opioid users, 33% reported having previously administered naloxone to an overdose patient [23]. Wakeman et al. found that of 137 previous opioid users, 90% had willingness to undergo a two-hour training for naloxone use [24]. Tobin et al. found similar results in a study design that included naloxone education with pre- and post-test and administration of a take-home naloxone kit. Of the 85 prior opioid users, 43 subsequently witnessed an overdose; 19 of the 43 were able to provide naloxone to the patient successfully [25]. We were unable to extrapolate from such studies whether street outreach volunteers would be as successful at administering naloxone as those who have lived experience with opioid use or naloxone administration. One limitation of our study is that we did not survey participants regarding any lived experience with opioid use or managing opioid overdose with naloxone. Additionally, our sample was a convenience sample of those present and willing to participate at the designated sites. While all but two of those who attended did elect to have their data included in the results (participated), the characteristics of those volunteers at individual sites who did not elect to attend the training are not known. The study design did not capture how many at each site were invited versus those who attended. The potential selection bias this might have on the study findings is not known.

Another limitation of our study was that we did not track participant use of naloxone following the training. We also did not assess participant concerns about naloxone administration, such as fears around repercussions of providing naloxone. Evans et al. found that of 198 participants in a naloxone training, only 45.5% understood the Good Samaritan Law, which offers legal protection to those providing immediate assistance to persons in danger [26]. We did not elicit feedback from the participants regarding their satisfaction or potential areas of improvement for the training.

Future studies could explore post-training utilization of naloxone by participants, retention of knowledge from the training, and outcomes in overdose victims receiving naloxone by study participants. Study design may also be improved by expanding the educational intervention by including hands-on training using a simulator mannequin. Surveying participants regarding barriers to naloxone use and addressing these concerns could also improve educational interventions around naloxone administration. Finally, to address the study limitation that we did not compare retention of knowledge by type of study site (street medicine clinic versus shelter versus soup kitchen), we could design a larger study with matched numbers of participants by training location type, and could also assess additional contributing factors such as educational background, and whether the participant had any prior medical training.

This data will be used to further improve naloxone education delivery to street medicine volunteers, and other street outreach team members in the community. Thus, this pilot project laid the groundwork for ongoing naloxone training. Following the completion of this study, the street medicine team continues to provide naloxone trainings at various sites that provide care to individuals experiencing homelessness. The cost of naloxone is now covered by county drug and alcohol funding. This study will help secure ongoing county funding for naloxone kits to be distributed to clinicians and volunteers who care for underserved high-risk populations such as people experiencing homelessness.

## Conclusions

In our study, the distribution of naloxone kits accompanied by an educational training addressing opioid overdose and naloxone administration was performed. Pre- and post-survey comparisons indicated improvement in responses across all age groups and by gender. Our findings give an example of how communities can use resources to effectively disseminate naloxone education and medication kits that serve high-risk community members such as those experiencing homelessness and substance use disorder.

## Appendices

Pre/Post Community Narcan Training Test

Code: ______________________________________________________

Age:

Gender (circle one):       Male       Female       Transgender       Nonbinary

Location of training (circle one):

Ripple Community Inc.

Safe Harbor Shelter

New Bethany Ministries

Trinity Soup Kitchen

Alliance Hall Soup Kitchen

Bethlehem Emergency Shelter

Allentown Warming Station

Other (please specify): ___________________________

1. True or false.

All opioids (e.g. Fentanyl, heroin, prescription pain pills) have the potential to cause physical dependence.

2. What is the leading cause of injury-related death in the United States?

a.       Alcohol overdose

b.       Falls

c.       Fire/burn

d.       Motor vehicle crashes

e.       Opioid overdose

3. What cause(s) a person to be at higher risk of an opioid overdose?

a.       Having a medical condition such as HIV, liver or lung disease

b.       Taking higher doses of an opioid

c.       Taking opioids with other sedating substances

d.       Taking opioids after reduced tolerance (detox or incarceration)

e.       All of the above

4. What is a symptom(s) of an opioid high?

a.       Blue fingernails or lips

b.       Constricted pupils

c.       Gurgling

d.       Unresponsive

e.       All of the above

5. What’s the first step in managing a suspected opioid overdose?

a.       Begin rescue breathing

b.       Call 911

c.       Give naloxone intra-nasally

d.       Lay person in recovery position

e.       Lay person on back

6. True or false.

Administration of naloxone should be continued every minute until patient is revived.

7. After the initial administration of naloxone, what is the next step?

a.       Begin hands-only cardiopulmonary resuscitation (CPR)

b.       Call 911

c.       Do not leave person

d.       Head tilt/jaw-thrust

e.       Lay person in recovery position

8. Which symptom(s) is indicative of opioid withdrawal?

a.       Body aches

b.       Dry skin

c.       Chest pain

d.       Sluggishness

e.       All of the above

9. Narcan is effective for reversing which of the following overdoses?

a.       Benzodiazepines

b.       Cocaine

c.       Fentanyl

d.       K2

e.       Methamphetamine

10. True or false.

The Good Samaritan Law only protects lay persons when responding to an opioid overdose and does not provide the person seeking medical attention after an overdose immunity from arrest.

## References

[1] (2018). CDC WONDER. https://wonder.cdc.gov/.

[2] Sweeney CT, Sembower MA, Ertischek MD, Shiffman S, Schnoll SH (2013). Nonmedical use of prescription ADHD stimulants and preexisting patterns of drug abuse. J Addict Dis.

[3] Carlson RG, Nahhas RW, Martins SS, Daniulaityte R (2016). Predictors of transition to heroin use among initially non-opioid dependent illicit pharmaceutical opioid users: a natural history study. Drug Alcohol Depend.

[4] O'Donnell JK, Halpin J, Mattson CL, Goldberger BA, Gladden RM (2017). Deaths involving fentanyl, fentanyl analogs, and U-47700 - 10 states, July-December 2016. MMWR Morb Mortal Wkly Rep.

[5] Ryan SA, Dunne RB (2018). Pharmacokinetic properties of intranasal and injectable formulations of naloxone for community use: a systematic review. Pain Manag.

[6] Chou R, Korthuis PT, McCarty D (2017). Management of suspected opioid overdose with naloxone in out-of-hospital settings: a systematic review. Ann Intern Med.

[7] Edwards ES, Gunn R, Kelley G, Smith A, Goldwater R (2015). AAPM 2015 Annual Meeting abstracts. Naloxone 0.4 mg bioavailability following a single injection with a novel naloxone auto-injector, EVZIO®, in healthy adults, with reference to a 1 mL standard syringe and intramuscular needle. Pain Med.

[8] Krieter P, Chiang N, Gyaw S (2016). Pharmacokinetic properties and human use characteristics of an FDA-approved intranasal naloxone product for the treatment of opioid overdose. J Clin Pharmacol.

[9] Avetian GK, Fiuty P, Mazzella S, Koppa D, Heye V, Hebbar P (2018). Use of naloxone nasal spray 4 mg in the community setting: a survey of use by community organizations. Curr Med Res Opin.

[10] Wampler DA, Molina DK, McManus J, Laws P, Manifold CA (2011). No deaths associated with patient refusal of transport after naloxone-reversed opioid overdose. Prehosp Emerg Care.

[11] You HS, Ha J, Kang CY (2020). Regional variation in states' naloxone accessibility laws in association with opioid overdose death rates-Observational study (STROBE compliant). Medicine (Baltimore).

[12] Guadamuz JS, Alexander GC, Chaudhri T, Trotzky-Sirr R, Qato DM (2019). Availability and cost of naloxone nasal spray at pharmacies in Philadelphia, Pennsylvania, 2017. JAMA Netw Open.

[13] Wagner KD, Valente TW, Casanova M (2010). Evaluation of an overdose prevention and response training programme for injection drug users in the Skid Row area of Los Angeles, CA. Int J Drug Policy.

[14] Buajordet I, Naess AC, Jacobsen D, Brørs O (2004). Adverse events after naloxone treatment of episodes of suspected acute opioid overdose. Eur J Emerg Med.

[15] Osterwalder JJ (1996). Naloxone--for intoxications with intravenous heroin and heroin mixtures--harmless or hazardous? A prospective clinical study. J Toxicol Clin Toxicol.

[16] Feldman BJ, Calogero CG, Elsayed KS (2017). Prevalence of homelessness in the emergency department setting. West J Emerg Med.

[17] Center for Behavioral Health Statistics and Quality (2015). 2014 National Survey on Drug Use and Health: Detailed Tables. Substance Abuse and Mental Health Services Administration.

[18] Dietze PM, Draper B, Olsen A (2018). Does training people to administer take-home naloxone increase their knowledge? Evidence from Australian programs. Drug Alcohol Rev.

[19] Lewis DA, Park JN, Vail L, Sine M, Welsh C, Sherman SG (2016). Evaluation of the overdose education and naloxone distribution program of the Baltimore Student Harm Reduction Coalition. Am J Public Health.

[20] Lockett TL, Hickman KL, Fils-Guerrier BJ, Lomonaco M, Maye JP, Rossiter AG (2018). Opioid overdose and naloxone kit distribution: a quality assurance educational program in the primary care setting. J Addict Nurs.

[21] Harris PA, Taylor R, Thielke R, Payne J, Gonzalez N, Conde JG (2009). Research electronic data capture (REDCap)--a metadata-driven methodology and workflow process for providing translational research informatics support. J Biomed Inform.

[22] Harris PA, Taylor R, Minor BL (2019). The REDCap consortium: building an international community of software platform partners. J Biomed Inform.

[23] Barocas JA, Baker L, Hull SJ, Stokes S, Westergaard RP (2015). High uptake of naloxone-based overdose prevention training among previously incarcerated syringe-exchange program participants. Drug Alcohol Depend.

[24] Wakeman SE, Bowman SE, McKenzie M, Jeronimo A, Rich JD (2009). Preventing death among the recently incarcerated: an argument for naloxone prescription before release. J Addict Dis.

[25] Tobin KE, Sherman SG, Beilenson P, Welsh C, Latkin CA (2009). Evaluation of the Staying Alive programme: training injection drug users to properly administer naloxone and save lives. Int J Drug Policy.

[26] Evans TI, Hadland SE, Clark MA, Green TC, Marshall BD (2016). Factors associated with knowledge of a Good Samaritan Law among young adults who use prescription opioids non-medically. Harm Reduct J.

